# Creation of immortalised epithelial cells from ovarian endometrioma

**DOI:** 10.1038/bjc.2012.26

**Published:** 2012-02-21

**Authors:** Y Bono, S Kyo, M Takakura, Y Maida, Y Mizumoto, M Nakamura, K Nomura, T Kiyono, M Inoue

**Affiliations:** 1 Department of Obstetrics and Gynecology, Kanazawa University Graduate School of Medical Science, 13-1 Takaramachi, Kanazawa, Ishikawa 920-8641, Japan; 2 Virology Division, National Cancer Research Institute, 5-1-1, Tsukiji, Chuo-ku, Tokyo 104-0045, Japan

**Keywords:** ovarian endometrioma, epithelial cells, immortalisation, progestin, oestrogen

## Abstract

**Background:**

Epithelial cells of endometriotic tissues are difficult to propagate *in vitro* as experimental material is scarce owing to their limited life span. However, there is an increasing concern regarding their malignant transformation in ovaries. The present study sought to generate their stable culture system.

**Methods and results:**

Purified epithelial cells isolated from ovarian endometriomas using microscopic manipulation were successfully immortalised by combinatorial transfection of human *cyclinD1*, *cdk4* and human telomerase reverse transcriptase (*hTERT*) genes, whereas the introduction of *hTERT* alone, or together with *cdk4*, was insufficient for immortalisation, leading to cellular senescence. We confirmed stable cytokeratin expression in the immortalised cells, proving their epithelial origin. These cells expressed progesterone receptor B and showed significant growth inhibition by various progestins. Oestrogen receptor (ER) expression was detected in these cells, albeit at low levels. Additional overexpression of ER*α* generated stable cells with oestrogen-dependent growth activation. Soft-agar colony formation assay and nude mice xenograft experiments demonstrated that these cells, even those with additional inactivation of *p53*, did not have transformed phenotypes.

**Conclusion:**

We for the first time generated immortalised epithelial cells from ovarian endometrioma that retained sex steroid responsiveness. These cells are invaluable tools not only for the consistent *in vitro* work but also for the study of molecular pathogenesis or carcinogenesis of endometriosis.

Endometriosis is a common gynaecological disorder associated with dysmenorrhoea, pelvic pain and subfertility and is a leading cause of disability and loss of productivity in women of reproductive age (Olive and Schwartz, 1993). Numerous studies have attempted to dissect the biology of endometriosis. These studies mainly use *in vitro* culture of stromal cells rather than culture of epithelial cells from endometriotic tissues, because the former cells are more easily and stably cultured for much longer periods than the latter cells (Noble *et al*, 1997; Gurates *et al*, 2002). In fact, it is difficult to culture endometriotic epithelial cells *in vitro*, because these cells lose their proliferative capacity during ongoing cultivation of primary cultures over several days. The inability of endometriotic epithelial cells to survive *in vitro* is an obstacle to gaining a better understanding of the biology of this disease. In particular, malignant change of endometriosis, especially of ovarian endometrioma, for which epithelial cells are exclusively responsible, has lately attracted considerable clinical attention (Kurman and Craig, 1972; McMeekin *et al*, 1995; Ness, 2003; Oral *et al*, 2003). There is therefore an urgent need to establish a stable system for the culture of endometriotic epithelial cells that can be used for research not only into the biology of endometriosis but also into its carcinogenesis.

There are two major barriers within epithelial cells that inhibit cell division under usual culture conditions: premature senescence and telomere-dependent senescence (Kiyono *et al*, 1998). The former is observed during early passage in primary culture and is caused by the activation of Rb that leads to cell cycle arrest, whereas the latter is found at a later stage of culture and is caused by telomere shortening after a considerable number of cell divisions. Inhibition of both Rb function and telomere shortening is therefore required for long-term culture of epithelial cells. In previous studies, we successfully established a stable system for the culture of primary endometrial epithelial cells, in which the human papillomavirus type 16 *E6/E7* genes and the human telomerase reverse transcriptase (*hTERT*) were introduced to inhibit Rb functions and to activate telomerase, respectively (Kyo *et al*, 2003). These immortal cells were not transformed but retained the original characteristics of endometrial epithelial cells, such as steroid responsiveness. Subsequent studies have demonstrated that overexpression of *cyclin D1* or *cdk4*, instead of HPV *E6/E7*, effectively inhibited Rb activity and might be an alternative method of overcoming premature senescence in primary epithelial cells of other origins (Ramirez *et al*, 2004; Sasaki *et al*, 2009).

In the present study, we sought to generate a stable culture of epithelial cells isolated from the ovarian endometriomas by the introduction of various genetic elements. These cells were successfully immortalised without generation of transformed phenotypes and were responsive to progestin and oestrogen. These cells are thus potentially useful as an experimental model for analysis of the mechanisms of steroid hormone functions as well as of carcinogenesis arising from ovarian endometrioma.

## Materials and methods

### Isolation and purification of human endometriotic glands

Human endometriotic tissue samples were obtained from a 27-year-old and a 44-year-old patient undergoing laparoscopic ovarian cystectomy as a treatment for ovarian endometrioma with written informed consent. Briefly, tissues were gently minced into small pieces (1–2 mm^3^) and were incubated for 1 h at 37 °C in a shaking water bath in 20 ml Hank's Balanced Salt Solution containing 0.2% collagenase type 3 (Washington Biochemical Corp., Lakewood, NJ, USA) and 1000 U of deoxyribonuclease I (Takara, Otsu, Japan). Epithelial glands were separated from stromal cells, blood cells and debris by serial filtration using narrow gauge sieves with apertures of 40–100 *μ*m. Individual glands on the bottom of the dishes were directly picked up one by one under a microscope, collected into Eppendorf tubes and seeded onto 24-well dishes for subsequent gene transfection by viral vectors. The use of clinical materials obtained with written informed consent was approved by the Institutional Research Ethics Committee.

### Vector construction and transfection

Viral construction and transfection of HPV16 *E6/E7* and *hTERT* have been previously reported (Kyo *et al*, 2003). Lentiviral vector plasmids were constructed by recombination using the Gateway system (Invitrogen, Carlsbad, CA, USA). Briefly, *hTERT*, *human cyclinD1* and *human mutant Cdk4* (Cdk4R24C: an inhibitor-resistant form of Cdk4 that was generously provided by Dr E Hara (The Cancer Institute of JFCR, Tokyo, Japan)) (Wölfel *et al*, 1995) were first recombined into entry vectors using the BP reaction (Invitrogen). These segments were then recombined with a lentiviral vector, CSII-CMV-RfA (a gift from Dr H Miyoshi (RIKEN BioResource Center, Tsukuba, Japan)) (Miyoshi *et al*, 1998), using the LR reaction (Invitrogen) to generate CSII-CMV-hTERT, -cyclinD1 and -hCDK4R24C. Production of recombinant lentiviruses with the vesicular stomatitis virus G glycoprotein was performed as described previously (Miyoshi *et al*, 1998). A dominant negative form of *p53* (*DN p53*) (Kiyono *et al*, 1994) was cloned into lentiviral vector plasmids by recombination using the Gateway system (Invitrogen). Oestrogen receptor *α* (ER*α*) overexpressing cells were established by lentiviral infection of the human ER*α* expression vector (pCMSCV-EM7bsd-hER*α*).

### Cell culture

Stably established endometriotic epithelial cells were maintained in DMEM supplemented with 10% fetal bovine serum in an atmosphere of 5% CO_2_ at 37 °C.

### Reverse transcriptase–PCR (RT–PCR)

Total RNA was isolated from cells using the RNeasy Mini Kit (Qiagen Sciences, Germantown, MD, USA), and the first-strand cDNA was synthesised from 1 *μ*g of total RNA by reverse transcription using Superscript II Reverse Transcriptase (Invitrogen) with random primers. Primer sequences and conditions for each gene are listed in Supplementary Table 1.

### Western blot analysis

Nuclear extracts from cells were prepared using the method of Schreiber *et al* (1989). Subsequently, 50 *μ*g of nuclear extracts were electrophoresed on a sodium dodecyl sulfate–polyacrylamide gel and transferred to polyvinylidene difluoride membranes. Membranes were blocked in TBST (150 mM NaCl, 20 mM Tris-Cl, pH 7.5 and 0.1% Tween) containing 5% nonfat dried milk and then incubated with specific antibodies against PR (H-190, dilution 1 : 1000, Santa Cruz Biotechnology, Santa Cruz, CA, USA) and actin (C-11, dilution 1 : 1000, Santa Cruz Biotechnology) followed by reaction with anti-rabbit IgG. Immunoreactive bands were visualised using the ECL detection system (GE Healthcare Biosciences, Pittsburgh, PA, USA), as suggested by the manufacturer.

### Immunocytochemistry and immunohistochemistry

Cells were cultured on LAB TEK chamber slides (Nalge Nunc International, Naperville, IL, USA) for 24 h, fixed with methanol. For antigen retrieval of CD10, the sections were heated by boiling in 10 mM citrate buffer, pH 6.0 for 20 min followed by cooling at room temperature for 20 min. Slides were incubated for 60 min at room temperature with the following mouse monoclonal antibodies and working dilutions: anti-pan-cytokeratin (4/5/6/8/10/13/18) (C11, dilution 1 : 500, Santa Cruz Biotechnology) and anti-CD10 (clone 56C6, dilution 1 : 80, Leica Microsystems Inc., Buffalo Grove, IL, USA). After incubation with an anti-mouse secondary antibody, immune complexes were visualised using the ABC-elite kit (Vector Laboratories Inc., Burlingame, CA, USA).

### *β*-gal assay

The *β*-gal assay was performed as previously described. Briefly, cells were fixed for 5 min at room temperature in 3% formaldehyde followed by incubation at 37 °C with senescence-associated *β*-gal stain solution containing 1 mg ml^−1^ of 5-bromo-4-chloro-3-indolyl *β*-D-galactoside (X-Gal), 40 mM citric acid/sodium phosphate, pH 6.0, 5 mM potassium ferrocyanide, 5 mM potassium ferricyanide, 150 mM NaCl and 2 mM MgCl_2_. After 6–12 h incubation, positive staining was confirmed using microscopy.

### *In vitro* growth assay

The proliferative activity of cells treated with progestins or oestrogen was examined by counting the cell number. Briefly, the cells were seeded at a density of 5–10 × 10^4^ cells per well in six-well flat-bottomed plates and were grown overnight in normal growth media at 37 °C. Cells that had been pre-incubated in normal growth media or in phenol red-free media containing charcoal-treated fetal bovine serum for 24 h were treated with 17*β*-estradiol (E2), 6*α*-methyl-17*α*-hydroxy-progesterone acetate (MPA), progesterone or dienogest at various concentrations. Ethanol was used as a vehicle control.

### Assay of aromatase activity

The aromatase activity of cells was assayed by detecting the formation of tritiated water from [1*β*-^3^H]-androstenedione (PerkinElmer Genetics, Bridgeville, PA, USA) as described (Shozu *et al*, 1997). We used a 4-h incubation for the experiment. Aromatase activity was expressed as the rate of incorporation of tritium into water per milligram of protein per 4 h of incubation.

### Anchorage-independent growth

A total of 2 × 10^5^ Ishikawa cells or immortalised cells were seeded onto 6-cm dishes containing 0.33% Noble agar in DMEM supplemented with 10% fetal calf serum on top of a 0.5% agar base in DMEM supplemented with 10% fetal calf serum. Colonies >0.2 mm were counted after incubation for 2 weeks.

### Mice xenograft experiments

Immortalised endometriotic epithelial cells were resuspended in growth media (10^8^ cells per ml) and were subcutaneously injected (0.1 ml) into the base of the bilateral flank of female BALB/c nu/nu mice (age range 7–9 weeks, SLC, Hamamatsu, Japan). Tumour growth was monitored weekly until confirmed tumours were visualised or at least for 2 months unless tumour formation was detected. All the experiments have been carried out with the ethical committee approval and meet the standards required by the UKCCCR guidelines (Workman *et al*, 2010).

## Results

### Generation of immortalised epithelial cells from ovarian endometrioma

**Figure 1:**
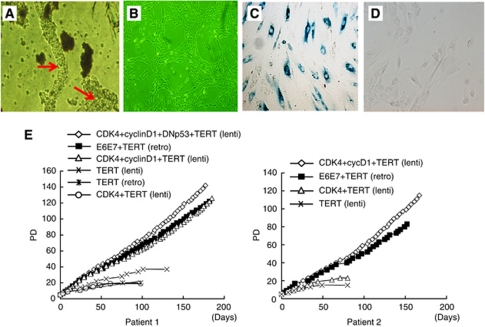
Morphological characteristics and proliferative life span of epithelial cells from ovarian endometrioma transfected with various genetic factors. (**A**) Phase contrast image of glandular clusters isolated from ovarian endometrioma tissues. Individual clusters were directly picked up, one by one, using microscopic manipulation and were transfected with various genetic factors. Glandular clusters are shown in arrows. (**B**) Phase contrast image of representative isolated clones (EMosis-CC /TERT-1 cells) cultured on plastic dishes are shown. (**C**) *β*-gal staining of cells from patient 1 transfected with *hTERT* alone (population doubling (PD): 20). (**D**) *β*-gal staining of EMosis-CC /TERT-1 cells (PD: 100). (**E**) The growth characteristics of transfected cells are represented as a growth curve. The genetic factors introduced are shown. Abbreviations: lenti=lentiviral vectors; retro=retroviral vectors.

Endometriotic tissues were collected from the surface epithelia of ovarian endometrioma of two patients, a 27-year-old (patient 1) and a 44-year-old (patient 2) patient who underwent laparoscopic ovarian cystectomy. These tissues were minced and digested in a collagenase solution. Endometriotic glands were then roughly isolated by serial filtration from the stromal cells, followed by direct pick-up, one by one, using microscopic manipulation (Figure 1A). Approximately 100 glands were individually seeded on the wells of plastic dishes and were infected with various combinations of lentiviral vectors for expression of *cyclinD1*, *cdk4*, *dominant negative p53* and *hTERT*. For comparative purposes, various combinations of retroviral vectors for expression of HPV16 *E6*, *E7* and *hTERT* (Kyo *et al*, 2003) were also introduced (Figure 1). Combinatorial transfection of at least three out of these genes successfully generated a total of five independent cell populations from the two patients that achieved >40 population doubling (PD). Two of these populations were transfectants harbouring the *cyclinD1*, *cdk4* and *hTERT* genes, two harboured the *E6*, *E7* and *hTERT* genes and the other population harboured *cyclinD1*, *cdk4*, dominant negative *p53* (*DN-p53*) and *hTERT* genes. Morphologically, all of these cells exhibited a small round shape that was compatible with an epithelial origin and formed a mesh-like structure on plastic dishes (Figure 1B). Introduction of the *hTERT* gene alone, or together with *cdk4,* generated cells from both patients that passed through 10 PD, but finally led to growth arrest at PD between 15–40, during which they exhibited morphological change to a large and flat shape. This phenomenon was determined to be senescence because these cells stained positive for the senescence-associated *β*-gal (Figure 1C). The cells derived from patient 1 and 2 that gained an extended life span, following the introduction of *cyclinD1*, *cdk4* and *hTERT* genes (and *DN-p53*), were named as EMosis-CC/TERT1 (and EMosis-CC/TERT/DNp53-1) and EMosis-CC/TERT2, respectively, and the cells into which HPV *E6/E7/TERT* were introduced were named as EMosis-E6/E7/TERT1 and EMosis-E6/E7/TERT2, respectively. These cells continued to grow for over 100 PD (Figure 1E), without any morphological change or senescence-associated *β*-gal staining (Figure 1D). To date, these cells have grown for over 200 PD and continue to grow. We have therefore concluded that these cells have gained immortal phenotypes. These findings indicate that co-expression of *cyclinD1* and *cdk4* are required in order to overcome the premature senescence of endometriotic epithelial cells and that these genes, combined with the expression of *hTERT,* are sufficient for their immortalisation, whereas the additional inactivation of *p53* is not necessarily required for immortalisation.

### Expression of epithelial markers and sex steroid receptors

**Figure 2:**
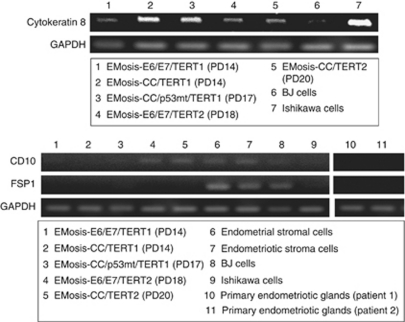
RT–PCR analysis of the expression of epithelial and stromal markers in immortalised epithelial cells from ovarian endometrioma. The expression of cytokeratin 8, CD10 and FSP1 in endometriotic epithelial cells immortalised by various genetic factors was examined using RT–PCR. Ishikawa and BJ cells were used as controls for epithelial and fibroblast cells, respectively. Primary endometriotic glandular cells without transfection, isolated from the ovarian endometrioma of patient 1 or 2, were used as negative controls for CD10 or FSP1 expression. GAPDH expression was assayed as a loading control.

**Figure 3:**
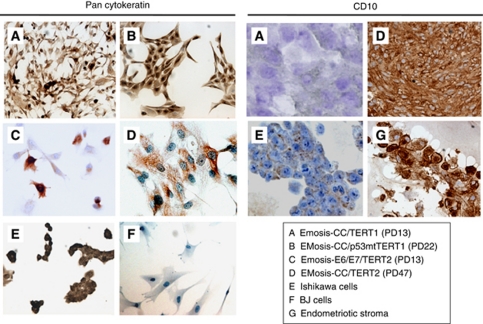
Immunocytochemical analyses of cytokeratin and CD10 expression in immortalised epithelial cells from ovarian endometrioma. The expression of cytokeratin and CD10 in endometriotic epithelial cells that were immortalised by various genetic factors and cultured on LAB TEK chamber slides was examined using immunocytochemistry. Ishikawa and BJ cells were used as controls for epithelial and fibroblast cells, respectively. Primary stromal cells without transfection, isolated from the ovarian endometrioma of another patient, were used as a positive control for CD10.

To confirm the origin of the immortalised cells, we next examined the expression of various epithelial and stromal cell markers using RT–PCR analysis. All isolated cells that had an extended life span expressed cytokeratin 8 mRNA, whereas mRNA expression of the stromal marker FSP1 was not observed (Figure 2). The mRNA expression of CD10, a marker that is characteristic of endometrial and endometriotic stromal cells (Sumathi and McCluggage, 2002; Toki *et al*, 2002), was not detected in EMOsis-CC/TERT1 or EMOsis-E6/E7/TERT1 cells but was detected in EMOsis-CC/TERT2 and EMOsis-E6/E7/TERT2 cells, whereas CD10 mRNA was not detected in primary endometriotic glands isolated from patients 1 and 2 (Figure 2). We also verified the epithelial origin of these clones using immunocytochemistry. As shown in Figure 3, all of these cells stained positive for pan-cytokeratin. However, although EMOsis-CC/TERT1 cells were negative for CD10, EMOsis-CC/TERT2 cells exhibited apparent CD10 staining.

**Figure 4:**
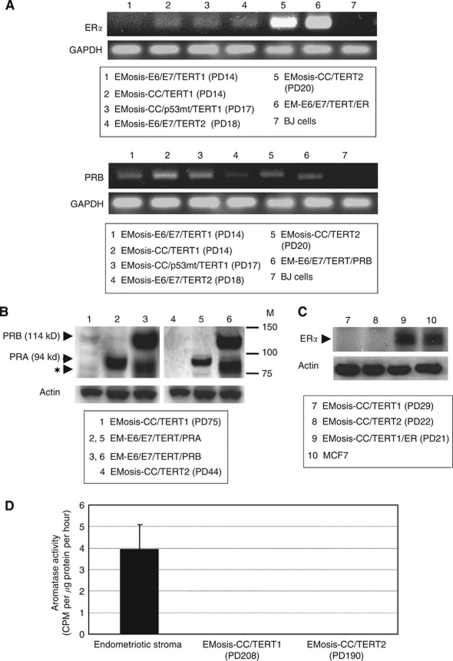
Sex steroid-receptor expression in, and aromatase activity of, immortalised epithelial cells from ovarian endometrioma. (**A**) RT–PCR analysis of expression of the oestrogen receptor *α* (ER*α*) or the progesterone receptor B (PRB). EM-E6/E7/TERT/ER cells are immortalised endometrial epithelial cells in which ER*α* cDNAs were stably transfected and were used as a positive control for ER*α*. EM-E6/E7/TERT/PRB cells are immortalised endometrial epithelial cells in which PRB cDNAs were stably transfected. Because our primer sets for PRB were designed to amplify the sequences containing *PRB* gene promoter in order to distinguish from PRA transcript, they can detect only intrinsic PRB mRNA but not extrinsic, overexpressed PRB mRNA that lacks promoter sequences. The weak PRB band in EM-E6/E7/TERT/PRB cells is therefore derived from intrinsic PRB. BJ cells were used as a negative control for ER*α* and PRB expression. GAPDH was used as a loading control. (**B**) Western blot analysis of expression of the progesterone receptor. EM-E6/E7/TERT/PRA or EM-E6/E7/TERT/PRB cells are immortalised endometrial epithelial cells in which PRA or PRB cDNAs were stably transfected and were used as a positive control for PRA or PRB expressions, respectively. Although EM-E6/E7/TERT/PRA cells showed a clear PRA band by western blotting (94 kDa), EM-E6/E7/TERT/PRB cells displayed two bands; one band was of the expected size of intact PRB (114 kDa); the other band was located just below the PRA band (identified by the symbol: ★) and was not a PRA band but a degraded PRB band, which was confirmed by another western blot analysis using a PRB-specific antibody (data not shown). EMosis-CC/TERT1 cells exhibited a weak, but distinct, PRB band but not a PRA band. M: protein weight marker. (**C**) Western blot analysis of expression of the ER. There was no detectable protein expression of ER*α* in EMosis-CC/TERT1 or EMosis-CC/TERT2 cells. EMosis-CC/TERT1/ER cells, generated by the introduction of ER*α* cDNA into EMosis-CC/TERT1 cells, were confirmed to have significant ER*α* expression. MCF7 cells were used as a positive control of ER*α* expression. (**D**) Analysis of aromatase activity using a tritiated water assay. Primary endometriotic stromal cells isolated from the ovarian endometrioma of another patient were used as a positive control of aromatase activity. Both EMosis-CC/TERT1 and EMosis-CC/TERT2 cells lacked aromatase activity.

We further investigated steroid-receptor expression in these cells using RT–PCR. ER*α* and progesterone receptor B (PRB) were expressed in all cell types that had an extended life span, except for EMOsis-E6/E7/TERT1 that lacked ER*α* expression (Figure 4A). Because expression of the PR isoform PRA, which has an 164 amino-acid deletion of PRB (Kastner *et al*, 1990), can not be discriminated from that of PRB using RT–PCR because of their identical gene sequences, we performed western blot analysis to distinguish the protein expression of these two PR isoforms. There was no detectable protein expression of PRA or even of PRB in any cell type except for EMOsis-CC/TERT1 that had detectable PRB protein expression (Figure 4B). The expression of ER*α* was not detected in these immortalised cells by western blot analysis (Figure 4C). These results were summarised in Supplementary Table 2. Aromatase expression is another factor that needs to be considered in relation to steroid-receptor expression. A tritiated water assay revealed that there was no detectable aromatase expression in any of the immortalised cells using assay conditions under which control primary endometriotic stromal cells exhibited significant aromatase activity (Figure 4D).

### Lack of transformed phenotypes in immortalised epithelial cells from ovarian endometrioma

**Figure 5:**
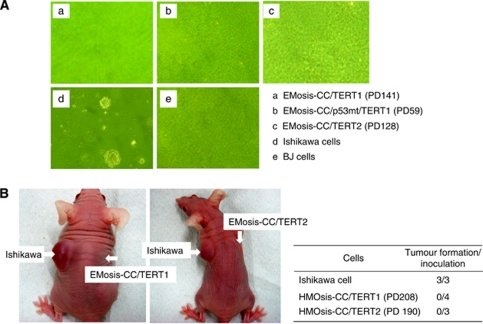
Analysis of the transformed phenotypes of immortalised epithelial cells from ovarian endometrioma. (**A**) Anchorage-independent growth was examined using a soft-agar colony formation assay. A total of 2 × 10^5^ Ishikawa cells or immortalised cells were seeded onto soft agar and colonies >0.2 mm were counted after incubation for 2 weeks. Ishikawa and BJ cells were used as a positive and negative control for colony formation, respectively. (**B**) *In vivo* growth was examined using a tumour formation assay in nude mouse. Immortalised epithelial cells from ovarian endometrioma were resuspended in growth media (10^8^ cells per ml) and were subcutaneously injected (0.1 ml) into the base of the bilateral flank of female BALB/c nu/nu mice (age range 7–9 weeks, SLC). Tumour growth was monitored weekly until confirmed tumours were visualised or at least for 2 months unless tumour formation was detected.

We next sought to determine whether these immortal cells had acquired a transformed phenotype. First, their growth properties were examined using a soft-agar colony formation assay. A total of 2 × 10^5^ cells were seeded on soft agar on 6-cm dishes and colonies with diameters >0.2 mm were counted after incubation for 2 weeks. Ishikawa or BJ cells were simultaneously examined as positive or negative controls, respectively. Although Ishikawa cells formed distinct colonies, neither the immortal epithelial cells nor the BJ cells formed colonies (Figure 5A). Tumourigenicity of these cells was also examined using nude mice. Control Ishikawa cells formed a subcutaneous tumour in mice 6 weeks after inoculation, but immortal epithelial cells were not able to form any tumour even 2 months after inoculation (Figure 5B).

### Responsiveness of immortalised epithelial cells from ovarian endometrioma to progestin and oestrogen

**Figure 6:**
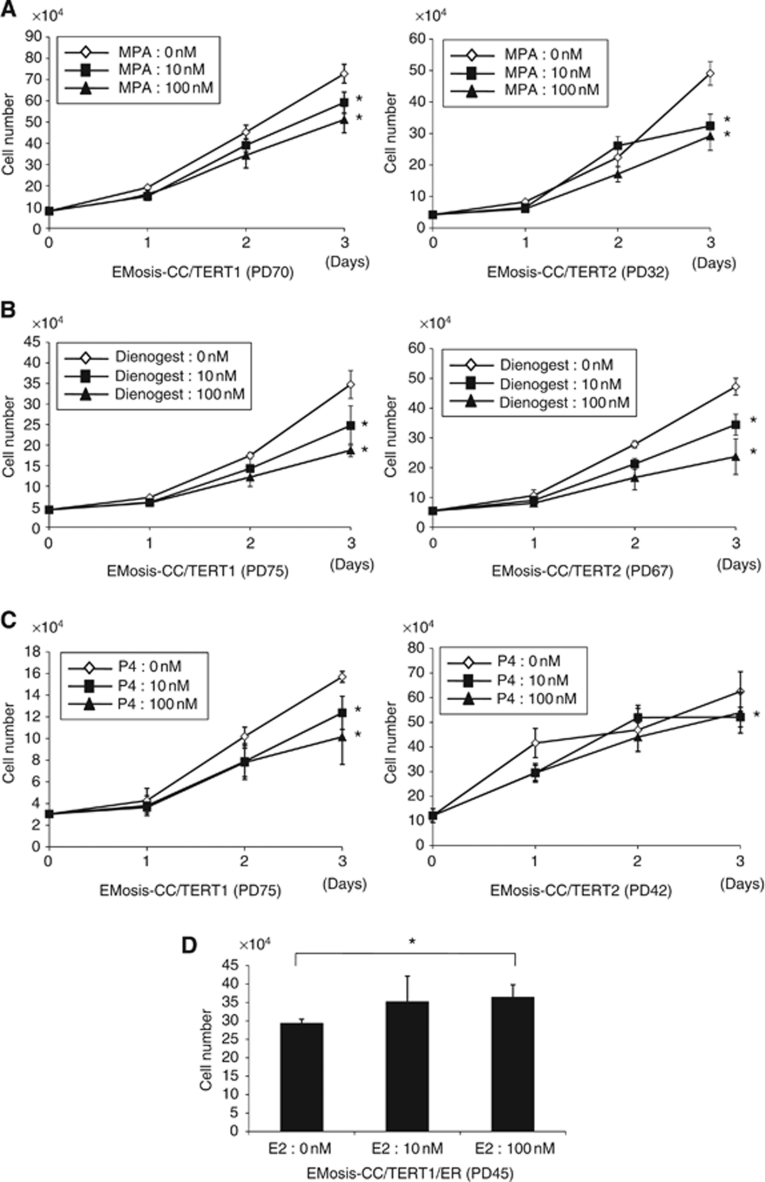
Effect of progestin and oestrogen on the growth of immortalised epithelial cells from ovarian endometrioma. EMosis-CC/TERT1 or EMosis-CC/TERT2 cells were seeded on six-well dishes and were treated with or without MPA (**A**), dienogest (**B**) or progesterone (P4) (**C**) at a concentration of 10 or 100 nM for the indicated number of days. Cell growth was monitored by counting cell numbers. Data are presented as means±s.d. of three independent experiments. ^*^P<0.05. (**D**) EMosis-CC/TERT1/ER cells were generated by the introduction of ER*α* cDNA into EMosis-CC/TERT1 cells and confirmed to have significant ER*α* expression (Figure 4C). EMosis-CC/TERT1/ER cells were seeded on six-well dishes and were treated with or without MPA estradiol (E2) at a concentration of 10 or 100 nM for the indicated number of days. Cell growth was monitored by counting cell numbers on day 5 after treatment. Data are presented as means±s.d. of three independent experiments. ^*^P<0.05.

We next examined the responsiveness of the immortalised epithelial cells to progestin. EMOsis-CC/TERT1 and EMOsis-CC/TERT2 cells were treated with MPA, dienogest or progesterone at a concentration of 1 or 100 nM for different time periods. Cell growth was then examined by counting cell numbers. Treatment with MPA or dienogest at a concentration of 10 or 100 nM significantly inhibited the growth of both cell types at 72 h (Figures 6A and B). Treatment with progesterone at 10 or 100 nM significantly inhibited the growth of EMOsis-CC/TERT1 at 72 h but only had a marginal effect on EMOsis-CC/TERT2 cells (Figure 6C). We performed these inhibitory experiments in growth media containing serum, considering clinical situations in which progestin is administrated *in vivo*. However, we also confirmed that progestin inhibited the growth of these cells in phenol red-free media containing charcoal-treated serum, although the extent of inhibition was less than that in normal growth media (data not shown), probably because of the cytostatic conditions of such media. These findings suggest that the immortalised epithelial cells preserved cell responsiveness to progestin.

We further examined the responsiveness of the immortalised epithelial cells to oestrogen. EMOsis-CC/TERT1 and EMOsis-CC/TERT2 cells were treated with 10 or 100 nM of 17*β* estradiol (E2) for different time periods. We failed to find any effect of E2 on the growth of either cell type (data not shown). This result was likely to be due to the low levels of ER*α* expression, which could only be faintly detected using RT–PCR. We therefore sought to overexpress ER*α* in EMOsis-CC/TERT1 cells via lentiviral introduction of ER*α* cDNA, and obtained ER*α*-overexpressing EMOsis-CC/TERT1 cells (EMOsis-CC/TERT1/ER). Sufficient expression of ER*α* in these cells was confirmed by western blot analysis (Figure 4C). The growth of EMOsis-CC/TERT1/ER cells was significantly activated by treatment with 17*β*-estradiol (E2) at a concentration of 100 nM (Figure 6D). Thus, we had successfully generated immortalised epithelial cells from ovarian endometrioma that still had the property of oestrogen or progestin responsiveness.

## Discussion

Although stromal cells in endometriotic tissues are easily isolated and grown under usual culture conditions, epithelial cells are hard to purify and propagate *in vitro*. This difficulty is mainly because of the rarity of epithelial cells in endometriotic tissues as well as to their shorter life span due to two barriers against their *in vitro* growth. To overcome these barriers, we first purified glandular fragments from endometriotic tissues that were treated with collagenase-based reagents via microscopic manipulation. We succeeded in immortalising endometriotic glandular cells through combinatorial introduction of the two genetic factors (*cyclin D1*/*cdk4*) that inhibit Rb functions together with *hTERT*. Special attention must be paid to contamination of the purified glandular fragments by stromal cells. This is because small amounts of stromal cells might possibly attach to the epithelial clusters providing a limitation to the purification of epithelial cells in glandular clusters. We therefore carefully judged whether the immortalised cells that we obtained were of epithelial origin. RT–PCR and immunocytochemical analyses confirmed the expression of epithelial markers in these cells. The introduction of *hTERT* alone, or the combination of *CDK4* and *hTERT,* failed to immortalise these cells. This result might provide further evidence of the epithelial origin of these cells, because stromal cells are usually immortalised by the introduction of *hTERT* alone (Kiyono *et al*, 1998; Morales *et al*, 1999). Indeed, Krikun *et al* (2004) confirmed immortalisation of endometriotic stromal cells by the introduction of *hTERT* alone. The requirement of two genetic factors that inhibit Rb function in addition to *hTERT* for cell immortalisation is consistent with observations in other epithelial cell types (Kiyono *et al*, 1998). Even the additional introduction of *DN-p53* failed to immortalise these cells, suggesting that they do not have high malignant potential, despite their invasive behaviour *in vivo*, which is reminiscent of cancer.

Although some studies showed that endometriotic tissues expressed PRB (Shen *et al*, 2008), others demonstrated that PRA was predominantly expressed and that PRB expression was low or absent (Attia *et al*, 2000; Wu *et al*, 2006). In the present study, one strain of the immortalised cells (EMosis-CC/TERT1) expressed PRB that was detectable using western blot analysis, whereas the other strain (EMOsis-CC/TERT2) did not. The reason why PRA was not detected in our western analysis remains unclear, but the expression might weaken or diminish during *in vitro* culture and/or the subsequent immortalisation step. Both EMosis-CC/TERT1 and EMosis-CC/TERT2 cells responded well to progestin, exhibiting significant growth retardation. It is of particular interest that, even though EMosis-CC/TERT2 cells only weakly expressed PRB, which was only detectable using RT–PCR, they were responsive to progestin, suggesting that such a low level of PRB expression was sufficient for a progestin effect. To our knowledge, this is the first demonstration of cultured epithelial cells from ovarian endometrioma that have stable progestin responsiveness. These cells are therefore a valuable tool for the study of progestin action in endometriosis. Progestin resistance is one of the characteristics of this disease (Vercellini *et al*, 2003; Bulun *et al*, 2006). However, some patients (approximately 50–70%) respond well to progestin-related agents, whereas others do not (Vercellini *et al*, 2003; Momoeda *et al*, 2009). Although the molecular mechanisms of this diversity among patients are not fully understood, some studies have indicated that the absence of, or decrease in, PR expression, possibly via promoter hypermethylation, has a key role in progestin resistance (Wu *et al*, 2006; Burney *et al*, 2007). We recently reported that fork head protein O1 (FOXO1) is a direct target of progestin for inhibiting endometrial epithelial growth (Kyo *et al*, 2011). Phosphorylated Akt has a critical role in this pathway by inhibiting FOXO1 activity, and the status of Akt is a predictor of progestin responsiveness in this cell type. It is therefore of interest to know whether a similar scenario of FOXO1 regulation by progestin exists in endometriotic epithelial cells, and this possibility is under investigation.

CD10 is a characteristic marker of both endometrial and endometriotic stromal cells (Toki *et al*, 2002, Sumathi and McCluggage, 2002). Although the endometriotic epithelial cells were isolated from patient 2 lacked CD10 expression before transfection, the EMosis-CC/TERT2 cells did express CD10 and, in addition, retained CK8 expression as demonstrated using RT–PCR (Figure 3). One possible explanation of these inconsistent results is that the contaminated stromal cells might have had a growth advantage during the immortalisation steps and therefore became the predominant population in the immortal cell culture. However, this possibility is not likely because these immortalised cells continued to express cytokeratin, which was confirmed using both RT–PCR and immunocytochemistry. Alternatively, the process of epithelial mesenchymal transition might be involved in this inconsistent expression of CD10, and this possibility is also under investigation. Recently, several novel mechanisms have been proposed to explain endometrial and endometriotic regeneration. One study showed that epithelial cells in the endometrium might have originated from stromal cells via cellular transdifferentiation (Garry *et al*, 2010). Another study suggested that both epithelial and stromal cells in eutopic/ectopic endometrium might arise from a common cell type with stem-like properties (endometrial stem/progenitor cells) (Maruyama *et al*, 2010). Both studies thus proposed a common origin of epithelial and stromal cells in eutopic/ectopic endometrium. The expression of the stromal marker (CD10) in endometriotic epithelial cells that was observed in the present study may be consistent with a hypothesis. We consider that this phenomenon is interesting and that it will be worthwhile to carry out further extensive analysis to uncover the origin of endometriotic cells.

Aromatase p450 is expressed in a number of tissues such as ovarian granulose cells, adipose tissue, skin fibroblasts and brain (Simpson *et al*, 1994). Aromatase catalyses the conversion of androstenedione to estrone, which is further converted to the potent oestrogen E2 by the enzyme 17*β*-hydroxysteroid dehydrogenase type 1. It is known that aromatase is absent in normal endometrium, whereas it is expressed in eutopic/ectopic endometrium in patients with endometriosis (Bulun *et al*, 1993; Noble *et al*, 1997; Zeitoun *et al*, 1999), where it increases local oestrogen production and thereby contributes to the development of this disease. Although it is well established that aromatase activity exists in stromal cells in endometriotic tissues, and this activity is well characterised, the presence and the role of aromatase in epithelial cells are largely unknown. Although a few immunohistochemical studies have shown aromatase expression in endometriotic epithelial cells (Kitawaki *et al*, 1997; Bulun *et al*, 2001), no study has confirmed this finding in *in vitro* cultures, probably because of the difficulty of *in vitro* culture of these cells. Our cell system gave us the opportunity to explore this point and demonstrated that the immortalised epithelial cells from ovarian endometrioma completely lacked aromatase activity, as determined using a tritiated water assay. Therefore, our result strongly supports the absence of aromatase activity in endometriotic epithelial cells.

Although ovarian endometrioma is a benign tumour, some endometriomas have been known to develop into malignant tumours with clear cell or endometrioid-type histology (Wölfler *et al*, 2005; Mandai *et al*, 2009). Although some genetic factors, including *PTEN* mutation, are known to be associated with ovarian cancers arising from endometrioma (Anglesio *et al*, 2010; Jones *et al*, 2010; Wiegand *et al*, 2010), the molecular mechanisms of carcinogenesis are largely unknown. The immortalised cells we established were found to lack transformed phenotypes. These cells might therefore be an ideal model for the study of carcinogenesis, in which candidate genetic factors can be introduced or knocked down, enabling the identification of genetic factors required for transformation. In particular, the ER*α*-expressing EMosis-CC/TERT1/ER cells appear to be suitable for such purposes. We are currently testing *in vivo* propagation of these cells in NOG mice together with endometriotic stromal cells to reconstitute endometriotic tissues with glandular structures, or hopefully, cancerous tissues after various genetic manipulations. Thus, these cells will be essential for the complete understanding of the multistep carcinogenesis of ovarian endometrioma and hopefully may be useful for identification of novel molecular therapeutic targets.
