# Supplementary material for: Creation of immortalised epithelial cells from ovarian endometrioma
Source: Br J Cancer. 2012 Feb 21;106(6):1205–13. doi: 10.1038/bjc.2012.26 (PMC3304406; doi:10.1038/bjc.2012.26)
Supplement: Supplementary Table 1 [file bjc201226x1.doc]

Supplementary Table 1 Primer sequence and conditions for RT-PCR

Gene Primer sequence Denaturation Annealing Cycle

ERα Forward 5’-AGACATGAGA GCTGCCAACC-3’ 95 °C (30 sec) 52 °C (60 sec) 32

Reverse 5’-GCCAGGCACATTCTAGAAGG-3’

PRB Forward 5’-TAGTGAGGGGGCAGTGGAAC-3’ 95 °C (30 sec) 57 °C (60 sec) 28

Reverse 5’-AGGAGGGGGTTTCGGGAATA -3’

Cytokeratin-8 Forward 5’-ACCCAGGAGAAGGAGCAGCT-3’ 95 °C (30 sec) 60 °C (60 sec) 25

Reverse 5’-CCGCC TAAGGTTGTTGATGT-3’

CD10 Forward 5’- TGTGGCCAGATTGATTCGTC -3’ 94 °C (60 sec) 62 °C (60 sec) 28

Reverse 5’-TTGTAGGTTCGGCTGAGGCT-3’

FSP1 Forward 5’-GAT GTGATGGTGTCCACCTT-3’ 95 °C (30 sec) 55 °C (60 sec) 28

Reverse 5’-ATTTCTTCCTGGGCTGCTTA-3’

GAPDH. Forward 5’-CTCAGACACCATGGGGAAGGTGA-3’ 95 °C (30 sec) 52°C (60 sec) 32

Reverse 5’-ATGATCTTGAGGCTGTT GTCATA-3’
