# Supplementary material for: Creation of immortalised epithelial cells from ovarian endometrioma
Source: Br J Cancer. 2012 Feb 21;106(6):1205–13. doi: 10.1038/bjc.2012.26 (PMC3304406; doi:10.1038/bjc.2012.26)
Supplement: Supplementary Table 2 [file bjc201226x2.doc]

Supplementary Table 2 Summary of the results of RT-PCR, Western and immunocytochemistry

PT-PCR

Cell lines Cytokeratin-8 CD10 FSP1 ERα PRB

EMosis-E6/E7/TERT1 + - - - +

EMosis-CC/TERT1 + - - + +

EMosis-CC/p53mt/TERT1 + - - + +

EMosis-E6/E7/TERT2 + + - + +

EMosis-CC/TERT2 + + - + +

Western

Cell lines PRA PRB ERα

EMosis-CC/TERT1 - + -

EMosis-CC/TERT2 - - -

Immunocytochemistry

Supplementary Table 2 Summary of the results of RT-PCR, Western and immunocytochemistry

Immunocytochemistry

Cell lines Pan-cytokeratin CD10

EMosis-CC/TERT1 + -

EMosis-CC/p53mt/TERT1 + not done

EMosis-E6/E7/TERT2 + not done

EMosis-CC/TERT2 + +
